# Genetic Determinants of Height Growth Assessed Longitudinally from Infancy to Adulthood in the Northern Finland Birth Cohort 1966

**DOI:** 10.1371/journal.pgen.1000409

**Published:** 2009-03-06

**Authors:** Ulla Sovio, Amanda J. Bennett, Iona Y. Millwood, John Molitor, Paul F. O'Reilly, Nicholas J. Timpson, Marika Kaakinen, Jaana Laitinen, Jari Haukka, Demetris Pillas, Ioanna Tzoulaki, Jassy Molitor, Clive Hoggart, Lachlan J. M. Coin, John Whittaker, Anneli Pouta, Anna-Liisa Hartikainen, Nelson B. Freimer, Elisabeth Widen, Leena Peltonen, Paul Elliott, Mark I. McCarthy, Marjo-Riitta Jarvelin

**Affiliations:** 1 Department of Epidemiology and Public Health, Imperial College London, London, United Kingdom; 2 Oxford Centre for Diabetes, Endocrinology and Metabolism, University of Oxford, Oxford, United Kingdom; 3 Wellcome Trust Centre for Human Genetics, University of Oxford, Oxford, United Kingdom; 4 The MRC Centre for Causal Analyses in Translational Epidemiology, Bristol University, Bristol, United Kingdom; 5 Institute of Health Sciences, University of Oulu, Oulu, Finland; 6 Biocenter Oulu, University of Oulu, Oulu, Finland; 7 Finnish Institute of Occupational Health, Oulu, Finland; 8 National Public Health Institute, Helsinki, Finland; 9 Biostatistics and Epidemiology Cluster, International Agency for Research on Cancer, Lyon, France; 10 London School of Hygiene and Tropical Medicine, University of London, London, United Kingdom; 11 Department of Child and Adolescent Health, National Public Health Institute, Oulu, Finland; 12 Department of Clinical Sciences/Obstetrics and Gynecology, University of Oulu, Oulu, Finland; 13 Center for Neurobehavioral Genetics, University of California Los Angeles, Los Angeles, California, United States of America; 14 The Jane and Terry Semel Institute for Neuroscience and Human Behavior, University of California Los Angeles, Los Angeles, California, United States of America; 15 Department of Psychiatry, University of California Los Angeles, Los Angeles, California, United States of America; 16 Finnish Genome Center, University of Helsinki, Helsinki, Finland; 17 Institute for Molecular Medicine (FIMM), University of Helsinki, Helsinki, Finland; 18 Broad Institute of Harvard and MIT, Cambridge, Massachusetts, United States of America; 19 Wellcome Trust Sanger Institute, Cambridge, United Kingdom; 20 Oxford NIHR Biomedical Research Centre, Churchill Hospital, Oxford, United Kingdom; The University of Queensland, Australia

## Abstract

Recent genome-wide association (GWA) studies have identified dozens of common variants associated with adult height. However, it is unknown how these variants influence height growth during childhood. We derived peak height velocity in infancy (PHV1) and puberty (PHV2) and timing of pubertal height growth spurt from parametric growth curves fitted to longitudinal height growth data to test their association with known height variants. The study consisted of N = 3,538 singletons from the prospective Northern Finland Birth Cohort 1966 with genotype data and frequent height measurements (on average 20 measurements per person) from 0–20 years. Twenty-six of the 48 variants tested associated with adult height (*p*<0.05, adjusted for sex and principal components) in this sample, all in the same direction as in previous GWA scans. Seven SNPs in or near the genes *HHIP*, *DLEU7*, *UQCC*, *SF3B4/SV2A*, *LCORL*, and *HIST1H1D* associated with PHV1 and five SNPs in or near *SOCS2*, *SF3B4/SV2A*, *C17orf67*, *CABLES1*, and *DOT1L* with PHV2 (*p*<0.05). We formally tested variants for interaction with age (infancy versus puberty) and found biologically meaningful evidence for an age-dependent effect for the SNP in *SOCS2* (*p* = 0.0030) and for the SNP in *HHIP* (*p* = 0.045). We did not have similar prior evidence for the association between height variants and timing of pubertal height growth spurt as we had for PHVs, and none of the associations were statistically significant after correction for multiple testing. The fact that in this sample, less than half of the variants associated with adult height had a measurable effect on PHV1 or PHV2 is likely to reflect limited power to detect these associations in this dataset. Our study is the first genetic association analysis on longitudinal height growth in a prospective cohort from birth to adulthood and gives grounding for future research on the genetic regulation of human height during different periods of growth.

## Author Summary

Family studies have shown that adult height is largely genetically determined. Identification of common genetic factors has been expedited with recent advances in genotyping techniques. However, factors regulating childhood height growth remain unclear. We investigated genetic variants of adult height for associations with peak height velocity in infancy (PHV1) and puberty (PHV2) and timing of pubertal growth spurt in a population based sample of 3,538 Finns born in 1966. Most variants studied associated with adult height in this sample. Of the 48 genetic variants tested, seven of them associated with PHV1 and five with PHV2. However, only one of these associated with both, and we found suggestive evidence for differential effects at different stages of growth for some of the variants. In this sample, less than half of the variants associated with adult height had a measurable effect on PHV1 or PHV2. However, these differences may reflect lower statistical power to detect associations with height velocities compared to adult height. This study provides a foundation for further biological investigation into the genes acting at each stage of height growth.

## Introduction

Height is a continuous complex trait which family and twin studies suggest is 80–90% heritable [1]–[3]. Recent genome-wide association (GWA) studies have found and replicated associations between common genetic variants from several genomic regions and adult height [4]–[7]. Each of the variants typically has only a small (∼0.2–0.6 cm/allele) effect on height [4]. Some of the SNPs identified lie in genes which are related to rare and severe monogenic syndromes impacting height in humans, or that can cause growth defects in mice when mutated [4].

Patterns of height growth vary from infancy to early adulthood and are controlled by a number of interacting mechanisms. The fastest gain is observed during the first year of life, followed by a period of slower growth, with another peak in puberty [8]. Longitudinal height growth analysis involves individual growth curve fitting and derivation of growth parameters from the fitted curves. Commonly derived biologically meaningful growth parameters include peak velocities at periods of fast growth and the timing of these peaks [8],[9]. The choice of periods of fast growth is based on prior knowledge of the biological regulation of height growth during these periods [10],[11].

Nutritional factors are known to have a considerable role in infancy whereas sex steroids and other hormones strongly regulate height growth in adolescence [12],[13]. This indicates that different biological pathways are involved in the augmentation of height at different stages of growth [10],[14]. We therefore expect that different patterns of genetic variation are associated with regulation of height growth at different stages, specifically at the two stages of fast growth: infancy and puberty. This hypothesis has been introduced before [15] but it has not yet been explored in population based genetic association studies.

This is the first study to evaluate the effect of genetic variants on different stages of height growth in a large prospective cohort from birth to adulthood. We assessed the associations between variants identified for adult height in GWA studies [4]–[7] and peak height velocities in infancy (PHV1) and puberty (PHV2) and two measures of timing of pubertal growth spurt: age at height growth spurt take-off (ATO) and age at peak height velocity in puberty (age at PHV2). These parameters were derived from longitudinal height growth measurements from birth until adulthood (on average 20 measurements per person) in the Northern Finland Birth Cohort 1966 (NFBC1966). The association between these variants and adult height in this sample was also assessed.

## Results

**Figure 1:**
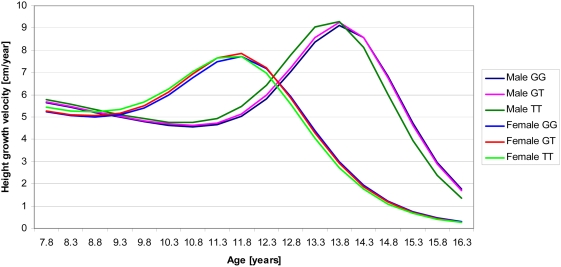
Mean-constant curves for height growth velocity between ages 8–16 y, estimated from the JPA-2 model (see Materials and Methods: Statistical Analyses), by sex and rs11107116 genotype (*SOCS2* gene, Table S2). Adult height increasing allele (T) is associated with higher PHV2 and earlier timing of pubertal height growth spurt.

Table 1 describes the growth outcomes in the NFBC1966. Males had a greater birth length, PHV1 and PHV2 while females had about two years earlier timing of pubertal growth spurt, measured by ATO and age at PHV2 (see Figure 1 which also shows how height velocity varies by age and sex between 8 and 16 years). The correlations between derived growth parameters and birth measures, adult height and body mass index (BMI) and age at menarche are as expected, showing internal consistency (Text S1, Table S1). For example, age at PHV2 had a correlation of r = 0.58 with age at menarche in girls and a weaker but still robust (p<0.0001) inverse correlation with BMI at 31 y in both sexes (r = −0.19 in girls, r = −0.17 in boys). Adult height was more strongly correlated with PHV1 (r = 0.45 in girls, r = 0.46 in boys) than PHV2 (r = 0.14 in girls, r = 0.09 in boys) whereas age at PHV2 did not have a correlation with adult height at p<0.05 level.

**Table 1:** Growth variables from longitudinal height data in NFBC1966 singletons with height SNP information, maximum N and mean (SD) given.

GROWTH VARIABLE	MALE (N = 1,763)	FEMALE (N = 1,775)	TOTAL (N = 3,538)
Birth weight [g]	3572 (520)	3455 (483)	3513 (505)
Birth length [cm]	50.8 (2.1)	50.0 (2.0)	50.4 (2.1)
Gestational age [weeks]	40.0 (1.9)	40.2 (1.8)	40.1 (1.9)
Ponderal index [kg/m^3^]	27.2 (2.4)	27.5 (2.4)	27.3 (2.4)
PHV1 (cm/year)	54.4 (3.2)	50.8 (3.9)	52.6 (4.0)
PHV2 (cm/year)	9.3 (1.4)	7.9 (1.1)	8.6 (1.5)
ATO (years)	11.2 (0.7)	9.3 (0.6)	10.3 (1.2)
Age at PHV2 (years)	13.9 (0.8)	11.7 (0.7)	12.8 (1.3)
Height at 31 years (cm)	178.3 (6.5)	164.7 (6.2)	171.4 (9.3)

PHV1 = peak height velocity in infancy from Reed1 model (see Materials and Methods: Statistical Analyses), PHV2 = peak height velocity in puberty from JPA-2 model, ATO = age at height growth spurt take-off, Age at PHV2 = age at peak height velocity in puberty.

Table 2 shows the associations between all SNPs, growth parameters and adult height from additive models per adult height increasing allele identified in previous studies. To assess age-dependent effects of the variants on growth velocity, the p-value for interaction between the SNP and age (puberty vs. infancy) on PHV is shown. The interaction analyses formally tested the hypothesis that different genetic variants are involved in height growth regulation at different stages of life. Due to a high correlation between ATO and age at PHV2 (Table S1), genetic associations for ATO are omitted from Table 2 but the main results are reported in the text. All the analyses were adjusted for sex and principal components (PCs; see Materials and Methods: Statistical Analyses) but not for socio-economic status (SES), birth length or gestational age since the additional adjustment for these variables did not essentially change the results. Table S2 shows further information on these SNPs, including SNP and gene information and allele frequencies. To assess statistical significance, we use p<0.05 significance level for adult height, PHV1, PHV2 and the age-SNP interaction on PHV. For the age at PHV2 and ATO association analyses and for sex-SNP interactions we use Bonferroni-corrected significance level of p<0.0011 level (see Materials and Methods: Statistical Analyses) because of weaker a priori evidence for the existence of the associations.

**Table 2:** Associations between SNPs and adult height, peak height velocity in infancy (PHV1) and puberty (PHV2) and age at PHV2.

Gene	SNP rs, allele^1^	Adult height	PHV1	PHV2	Int^2^	Age at PHV2
		Beta (SE(Beta)), p	Beta (SE(Beta)), p	Beta (SE(Beta)), p	p	Beta (SE(Beta)), p
*SF3B4/SV2A*	rs11205277, G	**0.43 (0.14), 0.0019**	**0.75 (0.18), 3×10^−5^**	**0.90 (0.43), 0.036**	0.19	−0.04 (0.02), 0.13
*LCORL* 3	rs6830062, T	**0.73 (0.22), 0.0010**	**0.74 (0.28), 0.0087**	0.88 (0.67), 0.19	0.38	0.00 (0.04), 0.89
*DLEU7*	rs3116602, T	**0.55 (0.15), 0.0003**	**0.60 (0.20), 0.0023**	0.31 (0.47), 0.51	0.12	0.01 (0.03), 0.73
*PPARD/FANCE*	rs4713858, G	0.17 (0.21), 0.41	0.45 (0.27), 0.091	0.01 (0.63), 0.99	0.16	0.01 (0.03), 0.67
*HIST1H1D*	rs10946808, A	**0.49 (0.13), 0.0002**	**0.44 (0.17), 0.0093**	−0.45 (0.40), 0.26	**0.0093**	0.00 (0.02), 0.84
*HHIP*	rs6854783, A	0.20 (0.14), 0.16	**0.41 (0.18), 0.025**	−0.33 (0.43), 0.44	*0.045*	0.01 (0.02), 0.59
*UQCC*	rs6060373, G	**0.69 (0.13), 2×10^−7^**	**0.41 (0.17), 0.016**	0.75 (0.41), 0.069	0.53	−0.02 (0.02), 0.42
*NHEJ1*	rs6724465, G	0.51 (0.26), 0.053	0.40 (0.34), 0.24	0.88 (0.80), 0.26	0.92	−0.05 (0.04), 0.21
*C6orf106*	rs2814993, A	**0.80 (0.17), 2×10^−6^**	−0.39 (0.23), 0.086	0.08 (0.52), 0.88	0.19	0.08 (0.03), 0.0057
*LCORL* 3	rs6842303, T	**0.39 (0.15), 0.67**	**0.38 (0.19), 0.044**	0.30 (0.45), 0.51	0.46	0.00 (0.02), 0.96
*SOCS2*	rs11107116, T	**0.47 (0.16), 0.0029**	−0.16 (0.21), 0.43	**1.60 (0.48), 0.0009**	**0.0030**	−0.06 (0.03), 0.015
*DOT1L*	rs12459350, G	0.20 (0.13), 0.13	0.09 (0.17), 0.59	**1.26 (0.41), 0.0021**	*0.047*	−0.03 (0.02), 0.17
*CABLES1*	rs4800148, A	0.24 (0.15), 0.12	0.38 (0.20), 0.056	**0.96 (0.47), 0.040**	0.75	−0.05 (0.03), 0.069
*SH3GL3* 3	rs2562785, T	0.07 (0.19), 0.44	−0.07 (0.25), 0.79	−0.92 (0.59), 0.11	0.27	0.03 (0.03), 0.28
*C17orf67*	rs4794665, A	**0.34 (0.13), 0.0094**	0.21 (0.17), 0.22	**0.82 (0.41), 0.046**	0.56	−0.02 (0.02), 0.46
*C6orf173*	rs4549631, C	0.18 (0.14), 0.19	0.17 (0.18), 0.34	0.71 (0.41), 0.085	0.69	−0.04 (0.02), 0.055
*PXMP3/PKIA*	rs7846385, C	−0.09 (0.16), 0.56	−0.06 (0.21), 0.76	0.40 (0.49), 0.41	0.40	−0.05 (0.03), 0.072
*HMGA2*	rs1042725, C	**0.47 (0.13), 0.0005**	0.13 (0.18), 0.48	−0.25 (0.42), 0.54	0.28	0.04 (0.02), 0.056
*ADAMTS17*	rs4533267, A	0.24 (0.15), 0.12	0.37 (0.20), 0.072	−0.44 (0.48), 0.36	0.068	0.04 (0.03), 0.10
*CDK6* 3	rs3731343, C	**0.29 (0.13), 0.028**	0.22 (0.17), 0.20	−0.69 (0.40), 0.084	0.029	0.04 (0.02), 0.050
*LIN28B*	rs314277, A	**0.45 (0.17), 0.0084**	0.06 (0.23), 0.81	0.33 (0.53), 0.53	0.97	0.04 (0.03), 0.15
*ACAN*	rs8041863, A	0.24 (0.14), 0.081	−0.01 (0.18), 0.96	0.20 (0.43), 0.64	0.68	−0.01 (0.02), 0.79
*SPAG17*	rs12735613, G	**0.41 (0.16), 0.0083**	0.15 (0.20), 0.46	0.51 (0.48), 0.28	0.85	−0.03 (0.03), 0.28
*CEP63*	rs10935120, G	−0.03 (0.15), 0.84	−0.22 (0.20), 0.27	0.30 (0.46), 0.52	0.25	−0.03 (0.03), 0.22
*ADAMTSL3* 3	rs10906982, A	**0.34 (0.14), 0.013**	−0.09 (0.18), 0.62	−0.18 (0.42), 0.66	0.96	0.00 (0.02) 0.90
*PTCH1*	rs10512248, G	0.11 (0.14), 0.42	0.00 (0.18), 0.98	0.28 (0.42), 0.51	0.54	0.00 (0.02), 0.84
*ZBTB38*	rs6440003, A	**0.61 (0.14), 7×10^−6^**	0.30 (0.18), 0.094	0.33 (0.42), 0.43	0.69	−0.03 (0.02), 0.24
*SCMH1*	rs6686842, T	0.16 (0.14), 0.25	−0.12 (0.18), 0.51	−0.13 (0.42), 0.76	0.97	0.00 (0.02), 0.96
*EFEMP1*	rs3791675, C	0.16 (0.16), 0.31	0.09 (0.20), 0.67	−0.23 (0.48), 0.63	0.53	0.00 (0.03), 0.91
*CDK6* 3	rs2282978, C	**0.31 (0.15), 0.038**	0.19 (0.20), 0.34	0.00 (0.46), 0.999	0.48	0.03 (0.03), 0.18
*CHCHD7*	rs9650315, G	**0.57 (0.21), 0.0056**	0.15 (0.28), 0.59	−0.06 (0.66), 0.93	0.81	0.01 (0.04), 0.77
*TRIP11* 3	rs8007661, C	0.01 (0.14), 0.94	0.06 (0.18), 0.76	0.57 (0.43), 0.18	0.48	0.01 (0.02), 0.72
*DNM3*	rs678962, G	**0.31 (0.15), 0.045**	−0.15 (0.21), 0.47	−0.20 (0.49), 0.68	0.69	−0.01 (0.03), 0.79
*TRIP11/FBLN5* 3	rs7153027, A	0.15 (0.13), 0.26	0.18 (0.17), 0.30	0.30 (0.40), 0.45	0.83	0.02 (0.02), 0.35
*ADAP2*	rs3760318, G	**0.37 (0.13), 0.0046**	−0.10 (0.17), 0.56	−0.27 (0.40), 0.51	0.80	0.01 (0.02), 0.75
*TBX2*	rs757608, A	**0.32 (0.15), 0.036**	0.12 (0.20), 0.55	−0.06 (0.47), 0.90	0.60	0.01 (0.03), 0.67
*BMP2*	rs967417, G	**0.41 (0.13), 0.0017**	0.24 (0.17), 0.16	0.05 (0.40), 0.90	0.49	−0.02 (0.02), 0.32
*BMP6*	rs12198986, A	0.15 (0.13), 0.26	0.13 (0.17), 0.43	−0.23 (0.39), 0.56	0.46	0.03 (0.02), 0.21
*RDBP, (LST1) NCR3/AIF1* 3	rs2844479, A	0.17 (0.15), 0.24	0.27 (0.19), 0.15	−0.01 (0.45), 0.98	0.46	−0.02 (0.02), 0.50
*RDBP/BAT3* 3	rs3130050, G	**0.62 (0.19), 0.0011**	−0.02 (0.24), 0.95	−0.64 (0.57), 0.26	0.55	0.00 (0.03), 0.88
*TNXB*	rs185819, C	−0.05 (0.13), 0.72	−0.11 (0.17), 0.72	0.12 (0.40), 0.77	0.52	−0.01 (0.02), 0.68
*HMGA1*	rs1776897, G	0.42 (0.28), 0.14	−0.11 (0.37), 0.77	0.19 (0.84), 0.82	0.84	−0.03 (0.05), 0.52
*GPR126* 3	rs6570507, G	**0.55 (0.15), 0.0002**	0.13 (0.19), 0.51	0.56 (0.46), 0.22	0.61	−0.01 (0.02), 0.64
*GPR126* 3	rs3748069, A	**0.55 (0.15), 0.0002**	0.22 (0.19), 0.26	0.68 (0.46), 0.14	0.69	−0.02 (0.02), 0.48
*AMZ1/GNA12*	rs798544, C	**0.27 (0.14), 0.046**	0.03 (0.18), 0.85	0.26 (0.42), 0.54	0.86	−0.01 (0.02), 0.71
*CDK6* 3	rs11765954, C	0.23 (0.15), 0.14	0.27 (0.20), 0.18	0.62 (0.47), 0.19	0.97	0.01 (0.03), 0.70
*PLAG1*	rs10958476, C	−0.21 (0.16), 0.19	−0.21 (0.21), 0.32	−0.52 (0.49), 0.28	0.90	0.04 (0.03), 0.16
*ZNF462*	rs4743034, A	**0.52 (0.16), 0.0016**	0.26 (0.21), 0.22	−0.14 (0.51), 0.78	0.15	0.04 (0.03), 0.17

All analyses are adjusted for sex and principal components. Results are sorted by effect sizes: ten largest for PHV1 at the top followed by remaining of ten largest for PHV2 and age at PHV2, followed by the remaining SNPs in arbitrary order. The SNPs with associations at p<0.05 significance level are highlighted for adult height, PHV1 and PHV2. Beta is expressed as the change in PHV in infancy and puberty [%], and as the change in age at PHV2 [year] per one adult height increasing allele.

1 Height increasing allele identified in GWAS (using the HapMap B35 + strand as the reference strand). The sign of adult height beta shows if the direction of effect was the same as in the three GWAS (+ = same, − = different).

2 Interaction p-value between SNP and age (puberty vs. infancy) on PHV, values at p<0.05 level are highlighted in italics and values at p<0.01 level in bold.

3 Genes with more than one SNP or SNPs close together in different genes. R^2^ between SNPs: *LCORL* 0.03, *SH3GL3/ADAMTSL3* 0.12, *RDBP, (LST1) NCR3/AIF1*/*RDBP/BAT3* 0.06 (all SNPs counted as separate signals); *CDK6 r^2^* 0.32–0.78, *TRIP11/FBLN5 r^2^* 0.72 and *GPR126 r^2^* 0.97 (counted as one signal per gene).

Based on LD in the NFBC1966, the 48 SNPs analysed represent 44 independent signals in 43 loci (see Materials and Methods: Genotyping of SNPs). Twenty-four of the 44 signals (corresponding to 26 of the 48 SNPs) associated (p<0.05) with adult height (Table 2). All of them had the same direction of effect as identified in GWA studies [4]–[6].

Seven SNPs in or adjacent to the genes *SF3B4/SV2A*, *LCORL*, *UQCC*, *DLEU7*, *HHIP* and *HIST1H1D* showed an association (p<0.05) with PHV1 (Table 2). All these SNPs except rs6854783 in *HHIP* were also associated with adult height in our study. All the SNP-PHV1 associations were in the same direction as SNP associations with adult height in the previous GWA studies and in the current study.

Five SNPs in or adjacent to the genes *SF3B4/SV2A*, *SOCS2*, *C17orf67*, *CABLES1* and *DOT1L* were associated at p<0.05 significance level with PHV2 (Table 2). Of these, three (related to *SF3B4/SV2A*, *SOCS2* and *C17orf67*) associated with adult height in our sample. All five associated in the same direction as with adult height in the previous studies and in our study. Two of the five (related to *SOCS2*, *CABLES1*) and two additional SNPs (related to *CDK6*, *C6orf106*) associated with timing of pubertal growth spurt (ATO and/or age at PVH2) at p<0.05. However, as we did not have a similar prior evidence for association with the timing of height growth spurt as for height velocities, we cannot declare even the strongest association with age at PHV2 (*C6orf106*, p = 0.0057) statistically significant after a Bonferroni correction for multiple testing.

Only SNP rs11205277 upstream of *SF3B4/SV2A* showed significant evidence for an association with both PHV1 and PHV2. SNP rs6830062 in *LCORL* had a similar effect size on PHV1 (beta 0.74%, 95% CI 0.19 to 1.21%) and PHV2 (0.88%, −0.44 to 2.17%) as had SNP rs6842303 in the same gene (PHV1 beta 0.38%, 0.01 to 0.76%, PHV2 beta 0.30%, −0.58 to 1.19%). The associations in *LCORL* were statistically significant for PHV1, but not PHV2, which may reflect inadequate power to detect association with PHV2.

Interaction between SNP and age on PHV was detected for four SNPs that had a main effect (p<0.05) on PHV1 and/or PHV2 (Table 2). For SNPs rs6854783 in *HHIP* and rs10946808 in *HIST1H1D* adult height increasing alleles increased PHV in infancy but not in puberty (p = 0.045 and 0.0093). SNPs rs11107116 (in *SOCS2*, see Figure 1 for velocity by genotype and age), and rs12459350 (*DOT1L*), showed an effect on PHV in puberty but not in infancy (p = 0.0030 and 0.047). Given the strong biological argument for differential effects at different ages [14], we considered the *SOCS2* and *HIST1H1D* interactions as suggestive and we also found a possible biological explanation for the *SOCS2* interaction. The *HHIP* and *DOT1L* interactions are borderline significant (just below p<0.05) but for the former there is also a possible biological explanation (see Discussion).

The interaction between sex and SNP effects on growth was investigated due to differences in growth parameters (see Table 1). We did not observe any statistically significant sex-SNP interactions on any of the outcomes after Bonferroni correction (at p<0.0011 level). The smallest p-value was observed for SNP rs2814933 (*C6orf106*) which could be associated with timing of pubertal growth spurt in males (age at PHV2 beta = 0.16 years) while in females there is no effect (age at PHV2 beta = −0.003 years; sex interaction p = 0.003). Due to only few interactions that were not significant after Bonferroni correction, the results are shown as sex-adjusted for all SNPs in Table 2.

## Discussion

Our study is the first genetic association study on longitudinal height growth in a large prospective cohort study from birth to adulthood. Frequent height measurements (on average 20 measurements/person) with exact measurement times were obtained from health clinic records. The data are representative of the original cohort and thus the population of Northern Finland (see Representativeness in Materials and Methods). Frequent height measurements from birth to adulthood are rarely available in large population based studies and this makes replication of the results challenging. Fitting similar models and deriving similar phenotypes across study populations would be required to ensure comparability of the results. This is, however, impossible without dense measurement points. One possibility in the future is to combine several smaller studies with dense height growth measurements for replication and meta-analysis.

The analyses show high internal quality of the parameters derived from the growth curve models based on their associations with observed birth measures, height, BMI and age at menarche. However, some assumptions had to be made to account for random variation associated with the derived parameters. The weighting of the SNP association analyses by the number of measurements per person within the age period in question assumes that the reliability of the growth data has a proportional relationship with the frequency of measurements taken within the age period, and that the measurement accuracy does not depend on the frequency of the measurements taken. Although these seem reasonable assumptions, they are difficult to verify using this data alone. Ideally the analyses would be weighted by the inverse of the variance attached to the phenotypes derived from the growth models. However, the variances for the derived outcomes could not be directly estimated from the models and we used weighting by the number of measurements as a proxy.

We chose a standard parametric approach to model longitudinal growth. This has the advantage of natural biological interpretability of the parameters obtained from the fitted models [9], and appeared to fit our data well. There are a number of alternative approaches, for instance smoothing or regression cubic splines; these are easy to fit but the interpretation of parameters poses challenges, as does the selection of the degree of smoothness to be enforced. We attempted to fit models based on cubic smoothing splines [16] to these data, but found the results difficult to interpret and sensitive to the number and location of knots selected, and therefore present only the results for the parametric growth models.

The results of the model comparison in the NFBC1966 for infant height were consistent with the model comparison on early weight growth in another study [17] in Congolese infants, where the Reed1 model showed the best fit. As far as we know, there are no published model comparisons for early height growth in other studies. For the whole period of growth from birth into adulthood, the superiority of the JPPS model over slightly simpler parametric models such as the Preece and Baines (PB1) and modified Shohoji and Sasaki (SSC) models has been described elsewhere [18], and was not tested in our data set. As expected, JPA-2 fitted better than JPPS into our data. The high correlation between ATO and age at PHV2 (Table S1) estimated from the JPA-2 model largely explains the similarities in the results between the two phenotypes. There was also a moderately high inverse correlation between PHV in puberty with the timing of pubertal height growth spurt. This may contribute to some overlap in the genetic association results, and has to be acknowledged in the interpretation of the results.

The power to detect an effect size of 0.46 cm per allele with adult height was 60% at level p<0.05 using MAF = 0.31 (average MAF among the 48 SNPs) and an additive genetic model. This contributes to the fact that almost half of the signals were not replicated in our study since the known height variants tested typically have a 0.2–0.6 cm per allele effect size.

The statistical power was slightly lower to identify similar effect sizes for PHV in infancy and puberty, and even lower to identify age-SNP interactions. Despite this, we found an interaction with a p-value of 0.0030 that together with a meaningful biological explanation gives suggestive evidence for a differential SNP effect by age. This SNP lies in *SOCS2* (*Suppressors of cytokine signalling 2*) which is a negative regulator of cytokine and cytokine hormone signalling via JAK/STAT pathways, and one of its functions is to influence growth and development through effects on growth hormone/IGF-1 signalling [19]. Estrogen has been shown to induce *SOCS2* expression in vitro, with a subsequent decrease in JAK-STAT signalling in response to growth hormone [20]. This potential role for *SOCS2* in the interplay between steroid hormones and growth, could explain the association we observe between *SOCS2* variation and growth velocity during puberty. The lack of association in early infancy could be explained by the fact that height growth is not yet dependent on growth hormone at that age [14]. Also, we found a possible biological explanation for the interaction (p = 0.045) for the SNP in *HHIP* (*Hedgehog interacting protein*), suggesting an effect on PHV in infancy but not in puberty. *HHIP* is a component of the hedgehog signal transduction pathway involved in embryogenesis and development [21]. This pathway influences the transcription of many target genes and is important for development of many tissues and organs. It is important in early embryogenesis and cell proliferation, including limb and central nervous system development [21],[22]. Therefore it seems plausible that variants in *HHIP* would only play a role in early infancy but not in puberty. However, since the *HHIP* interaction does not appear to be very strong in our data, this result needs replication.

To summarise, our results show that nearly half of the genetic variants associated with adult height in this sample had a *measurable* effect on PHV in infancy or puberty. Only one variant was associated with PHV in both infancy and puberty. We found suggestive evidence that the associations of some of the variants may be age-dependent. The majority of signals associated with growth parameters in this study lie close to genes that are involved in recognised growth and development pathways, or have a potential role in growth through an effect on gene expression or regulation (e.g. cell proliferation, bone formation and growth hormone signalling pathways). Heritability of adult height is well documented [23]–[25] but heritability of height velocity at different stages of growth is less well established, although some estimates have been provided from family and twin studies [26]. Our study is the first population based genetic study of longitudinal height growth, and provides an insight into how height in humans may be regulated by its genetic determinants during different periods of growth.

## Materials and Methods

### Samples

Women expected to give birth in 1966 in the provinces of Oulu and Lapland were invited to participate in the Northern Finland Birth Cohort of 1966 (NFBC1966). Data were collected in pre-natal clinics and at birth (e.g. birth weight, length, n = 12,058 live births) [27],[28]. Details of the measurement protocols are published elsewhere [27],[29]. Additional data were collected via health clinics at age 1 y (n = 10,821), postal questionnaire at 14 y (n = 11,010) and 31 y (n = 8,690), and further data on postnatal growth were obtained from communal health clinics.

On average 20 height measurements per person were obtained from birth until adulthood (most between ages 0–16 y). About 25% of the records requested had gone missing over the years or could not be obtained. The final number of individuals with growth data and DNA samples was N = 4,311. The number of singletons with growth and genotype data after exclusions explained in the Statistical Analyses was N = 3,538. The measurement times were chosen by national recommendations but there was some variation between individuals.

**Figure 2:**
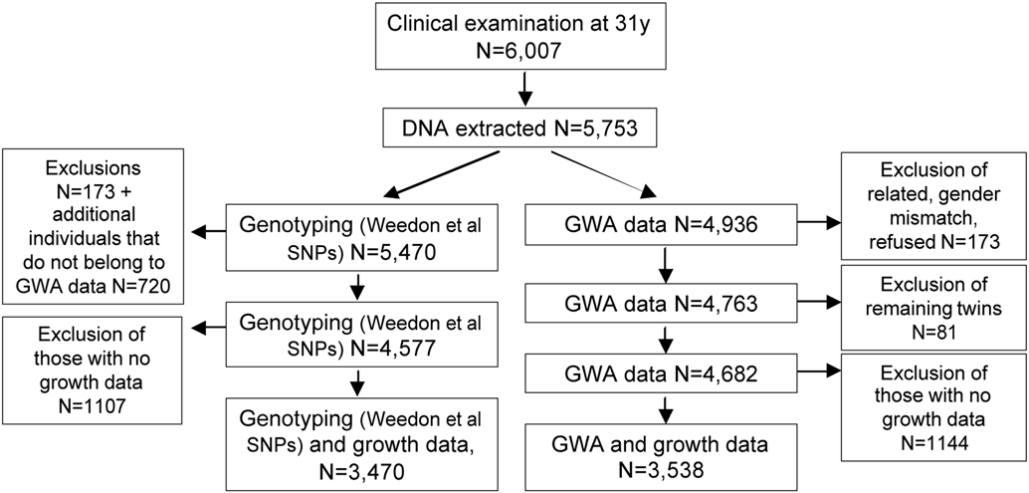
Flow chart of genotyping strategy for the genetic association study on height growth in the NFBC1966. The left arm shows genotyping done separately for 19 SNPs from Weedon et al, 2008 [4]; the right arm shows the GWA route to identify further 29 SNPs [5],[6]. The maximum number in final analyses was 3,538 with both growth and genotype information.

Individuals still living in northern Finland or the Helsinki area at 31 y were invited to a clinical examination (n = 6,007 attended). Anthropometric measurements, samples for biochemical assays and for DNA extraction and genotyping (n = 5,753) were collected (Figure 2). Informed consent for the use of the data including DNA was obtained from all subjects. The present study was approved by ethics committees in Oulu and Oxford universities in accordance with the Declaration of Helsinki.

### Genotyping of SNPs

Nineteen SNPs that associated with adult height in Weedon et al, 2008 [4] or their proxies were genotyped using DNA collected as part of the NFBC1966 cohort at age 31 y. 5,470 DNA samples were available; maximum 4,577 were included in the final analyses due to the exclusions explained in the Statistical Analyses (see also Figure 2). Genotyping was conducted using TaqMan SNP genotyping assays (Applied Biosystems, Foster City, California). PCRs were carried as recommended in the assay literature and genotypes derived from a 7900HT Sequence Detection System plate reader (Applied Biosystems, Foster City, California). Twelve positive samples and twelve negative wells were used as part of the quality control protocol. Genotyping results were checked to ensure the allele frequencies were in HWE. A full plate (384) was duplicated for the purposes of quality control. The duplication error rate was calculated as the number of (genotypes disagreed/number of samples duplicated)/2. For most assays the duplication error rate was zero with no discrepancies between the results. There were four assays where one or two samples were discrepant between the two sets of genotyping (where approximately 340 samples were duplicated on both plates).

Additional 29 SNPs that associated with height in two other publications [5],[6] or their proxies were obtained from a genome-wide scan for the NFBC1966 (original, detailed description in [30]) using Illumina's HumanCNV370-Duo DNA Analysis BeadChip. All these SNPs were directly genotyped (no imputed genotypes were used). Individuals who refused data delivery to collaborating units or had a gender mismatch between genotype and phenotype data were excluded from all analyses. Of those who had relatedness coefficient >0.20 (twins, half-siblings), the one with less complete genotype data was excluded at this stage. The number of exclusions in total was 173, leaving N = 4,763. Further exclusions explained in the Statistical Analyses reduced the final N to 4,682 with genome wide data. Figure 2 shows the identification of SNPs for our analyses, i.e. two “arms”, the one for genotyping done separately for NFBC1966 and the other for identification of SNPs from the NFBC1966 GWA data.

Basing our analyses on the sub-sample with GWA data enabled us to correct for cryptic relatedness and population structure via PC analysis (see Statistical Analyses). The genetic association results in the full genotyped sample and the sub-sample with GWA were not materially different. Since Weedon et al, 2008 [4] used a different platform (Affymetrix 500 K chip) for genotyping, we could not directly obtain all the SNPs they identified from our GWA data. We could have imputed them but preferred to use directly genotyped SNPs.

The 48 SNPs from the recent GWA scans [4]–[6] or their proxies represent 43 separate loci (*TRIP11*, *GPR126*, *LCORL* with two SNPs and *CDK6* with three SNPs in or near each). The SNPs in or near *TRIP11*, *GPR126* and *CDK6* were in high LD with each other in the NFBC1966 sample (r^2^ = 0.32–0.97) and therefore were counted as one signal per gene, giving the total number of 44 independent (r^2^≤0.12) signals within 43 loci.

### Statistical Analyses

PC analysis was applied in the genome-wide scan sample of N = 4,763 to characterize the genetic distances between persons within the sample. The first 20 PCs were analysed in association with birth length, adult height, PHV1, PHV2 and age at PHV2 by sex. In addition to first five PCs, the PCs that were associated with one or more of the growth outcomes in either sex (PCs 11, 13 and 15) were adjusted in all SNP association analyses to control for population structure (see the recommendation by Novembre and Stephens [31]). Additional adjustment for socio-economic status at birth (SES) did not change the results essentially and was not applied. Unpublished data on this cohort show that adjustment for PCs partly corrects for SES in the (genome-wide) analysis of adult height due to a correlation between SES and some of the PCs. Adjustment for PCs also corrects for parental geographic location.

Sex was adjusted in all SNP association analyses (sex-interactions explored and reported separately). All remaining twins were removed from the analyses, leaving 4,682 for genetic analyses. Number was reduced further due to missing data in the phenotypes, e.g. for final height N = 4,677 and for growth data maximum N = 3,538 (Figure 2) which was further reduced depending on the minimum number of measurement points required for analysis at certain age windows, as explained in Text S1.

This study is hypothesis based since it utilises prior information from GWA studies and can consequently be likened to candidate gene studies. Therefore statistical significance was considered at p<0.05 level for the SNP associations on adult height, PHV1 and PHV2 and the age-SNP interaction on PHV. Since we do not have similar prior information for the timing of height growth spurt, we only declare statistical significance at p<0.0011 level for ATO and age at PHV2. This level is based on Bonferroni correction considering 44 independent signals. Previous GWA studies found no evidence for sex-SNP interactions on adult height, although sex is an important determinant of growth and adult height [4]–[6]. We test sex-SNP interactions on each outcome but due to the absence of prior evidence for interactions use Bonferroni correction (p<0.0011 level) for assessing their statistical significance.

### Association Analysis of Genetic Variants and Growth Parameters

Description of growth curve fitting and derivation of growth parameters from the fitted curves is described in Text S1. The derived parameters from the Reed1 [32] and Jolicoeur-Pontier-Abidi-2 (JPA-2) [33] models were used separately as outcomes in the SNP association analysis. Due to skewness, natural logarithmic transformation was used for PHV1 and PHV2. To account for the random variation attached to the derived growth parameters, the association analyses were weighted by the number of measurements per person within the age period in question (infancy: 0–24 months, puberty: 8–16 years for girls, 9–17 years for boys). A regression model assuming an additive genetic effect was fitted between each SNP and each growth parameter, adjusted for sex and PCs. Additionally, the same analyses were run with sex-SNP interaction included. Preliminary analyses showed that adjusting additionally for birth length and gestational age does not essentially change the results, and this adjustment was not done. Results are reported per one allele increase in the genotype, the reference allele being the height decreasing allele in the previous GWA studies. SAS (version 9.1.3.) was used for all the association analyses of genetic variants and growth parameters.

In addition, the interaction between SNP effects and age (infancy vs. puberty) on peak height velocity (PHV) was tested. This was necessary as especially in the context of low power; finding that some SNPs are statistically significantly associated with PHV at one age and not the other does not automatically indicate different pattern of associations between these ages. Since PHV is much higher in infancy than in puberty, PHV Z-scores were calculated from the log-transformed PHV variables at each age to unify their scale. The data from infancy and puberty were combined into a single data set where most individuals had PHV values for both ages, i.e. two records per person, age indicator variable referring to the time when PHV was estimated (0 = infancy, 1 = puberty). A mixed model for repeated measures that takes into account the within-person correlation in the outcome values was chosen. The mixed model was fitted between each SNP and PHV Z-score without pre-defined covariance structure for the error matrix (type = unstructured), with SAS PROC MIXED (version 9.1.3.). Age was included into the model as a binary variable (0 = infancy, 1 = puberty) and the age-SNP interaction was tested. The analysis was weighted by the number of measurement points at the age window in question (on average 7–8 measurements per person at both ages). The model was additionally adjusted for sex and PCs.

### Power Calculations

Statistical power was 60% to detect a per allele effect size of 6.0% SD (0.24 cm/year) for PHV1, 6.6% SD (0.10 cm/year) for PHV2, and 4.9% SD (0.46 cm) for adult height, assuming a MAF of 0.31, which was the average among the 48 SNPs, additive genetic model and significance threshold p<0.05. For comparison, we had 80% statistical power to detect a per allele effect size of 7.6% SD (0.30 cm/year) for PHV1, 8.4% SD (0.13 cm/year) for PHV2, and 6.2% SD (0.58 cm) for adult height with the same assumptions. Quanto (version 1.2.3.) [34] was used for the power calculations.

### Representativeness

The sub-sample that attended the clinical examination at age 31 y is adequately representative of the NFBC1966 in terms of gender and socio-economic indicators at birth and at age 31 y [35]. Even better representativeness was observed when the sub-group with growth data and height SNP information (N = 3,538) was compared with attendees of clinical examination who did not have this information available (N = 2,469). In this comparison, men had data available slightly more often than women (61% vs. 57%). There were no differences regarding unemployment history or education (data available for 58–60% in all groups). There were small differences between social classes at birth (data available for 56–62% in all groups). At age 31 y, other social classes had more often data available than farmers (57–62% vs. 51%), but it has to be noted that this may be explained by random variation since the farmers group at 31 years is small (N = 214).

## Supporting Information

Table S1 Spearman correlation coefficient between growth parameters and growth measures at birth and in adulthood.
(0.06 MB DOC)

Table S2 Summary of the SNPs genotyped in the NFBC1966.
(0.26 MB DOC)

Text S1 Description of growth modelling methods, derived growth parameters and their correlations with other growth measures.
(0.05 MB DOC)

## References

[1] Perola M, Sammalisto S, Hiekkalinna T, Martin NG, Visscher PM (2007). Combined Genome Scans for Body Stature in 6,602 European Twins: Evidence for Common Caucasian Loci.. PLoS Genet.

[2] Silventoinen K, Sammalisto S, Perola M, Boomsma DI, Cornes BK (2003). Heritability of Adult Body Height: A Comparative Study of Twin Cohorts in Eight Countries.. Twin Res.

[3] Macgregor S, Cornes B, Martin N, Visscher P (2006). Bias, precision and heritability of self-reported and clinically measured height in Australian twins.. Hum Genet.

[4] Weedon MN, Lango H, Lindgren CM, Wallace C, Evans DM (2008). Genome-wide association analysis identifies 20 loci that influence adult height.. Nat Genet.

[5] Gudbjartsson DF, Walters GB, Thorleifsson G, Stefansson H, Halldorsson BV (2008). Many sequence variants affecting diversity of adult human height.. Nat Genet.

[6] Lettre G, Jackson AU, Gieger C, Schumacher FR, Berndt SI (2008). Identification of ten loci associated with height highlights new biological pathways in human growth.. Nat Genet.

[7] Sanna S, Jackson AU, Nagaraja R, Willer CJ, Chen W-M (2008). Common variants in the GDF5-UQCC region are associated with variation in human height.. Nat Genet.

[8] Molinari L, Gasser T, Hauspie RC, Cameron N, Molinari L (2004). The human growth curve: distance, velocity and acceleration.. Methods in Human Growth Research.

[9] Hauspie RC, Molinari L, Hauspie RC, Cameron N, Molinari L (2004). Parametric models for postnatal growth.. Methods in Human Growth Research.

[10] Cameron N (2002). Human growth and development.

[11] Thomis MA, Towne B (2006). Genetic determinants of prepubertal and pubertal growth and development.. Food Nutr Bull.

[12] Liu Y, Jalil F, Karlberg J (1998). Risk factors for impaired length growth in early life viewed in terms of the infancy-childhood-puberty (ICP) growth model.. Acta Paediatr.

[13] Veldhuis JD, Roemmich JN, Richmond EJ, Rogol AD, Lovejoy JC (2005). Endocrine Control of Body Composition in Infancy, Childhood, and Puberty.. Endocr Rev.

[14] Tse WY, Hindmarsh PC, Brook CGD (1989). The infancy-childhood-puberty model of growth: Clinical aspects.. Acta Paediatr Scand.

[15] Sammalisto S (2008). Search for Genetic Variants Influencing Human Height.

[16] Hastie TJ, Tibshirani RJ (1990). Generalized Additive Models.

[17] Simondon KB, Simondon F, Delpeuch F, Cornu A (1992). Comparative study of five growth models applied to weight data from congolese infants between birth and 13 months of age.. Am J Hum Biol.

[18] Ledford AW, Cole TJ (1998). Mathematical models of growth in stature throughout childhood.. Ann Hum Biol.

[19] Greenhalgh CJ, Alexander WS (2004). Suppressors of cytokine signalling and regulation of growth hormone action.. Growth Horm IGF Res.

[20] Leung KC, Doyle N, Ballesteros M, Sjogren K, Watts CK (2003). Estrogen inhibits GH signaling by suppressing GH-induced JAK2 phosphorylation, an effect mediated by SOCS-2.. Proc Natl Acad Sci U S A.

[21] Chuang P-T, McMahon AP (1999). Vertebrate Hedgehog signalling modulated by induction of a Hedgehog-binding protein.. Nature.

[22] Jeong J, McMahon AP (2005). Growth and pattern of the mammalian neural tube are governed by partially overlapping feedback activities of the hedgehog antagonists patched 1 and Hhip1.. Development.

[23] Pan L, Ober C, Abney M (2007). Heritability estimation of sex-specific effects on human quantitative traits.. Genet Epidemiol.

[24] Pilia G, Chen W-M, Scuteri A, Orru M, Albai G (2006). Heritability of Cardiovascular and Personality Traits in 6,148 Sardinians.. PLoS Genet.

[25] Silventoinen K, Haukka J, Dunkel L, Tynelius P, Rasmussen F (2008). Genetics of Pubertal Timing and Its Associations With Relative Weight in Childhood and Adult Height: The Swedish Young Male Twins Study.. Pediatrics.

[26] Silventoinen K, Pietilainen KH, Tynelius P, Sorensen TI, Kaprio J (2008). Genetic regulation of growth from birth to 18 years of age: the Swedish young male twins study.. Am J Hum Biol.

[27] Rantakallio P (1988). The longitudinal study of the northern Finland birth cohort of 1966.. Paediatr Perinat Epidemiol.

[28] Bennett AJ, Sovio U, Ruokonen A, Martikainen H, Pouta A (2008). No evidence that established type 2 diabetes susceptibility variants in the PPARG and KCNJ11 genes have pleiotropic effects on early growth.. Diabetologia.

[29] Bennett AJ, Sovio U, Ruokonen A, Martikainen H, Pouta A (2004). Variation at the insulin gene VNTR (variable number tandem repeat) polymorphism and early growth: studies in a large Finnish birth cohort.. Diabetes.

[30] Sabatti C, Service SK, Hartikainen AL, Pouta A, Ripatti S (2009). Genome-wide association analysis of metabolic traits in a birth cohort from a founder population.. Nat Genet.

[31] Novembre J, Stephens M (2008). Interpreting principal component analyses of spatial population genetic variation.. Nat Genet.

[32] Berkey CS, Reed RB (1987). A model for describing normal and abnormal growth in early childhood.. Hum Biol.

[33] Jolicoeur P, Pontier J, Abidi H (1992). Asymptotic models for the longitudinal growth of human stature.. Am J Hum Biol.

[34] Gauderman WJ, Morrison JM (2006). Quanto 1.1: A computer program for power and sample size calculations for genetic-epidemiology studies.. http://hydra.usc.edu/gxe.

[35] Sovio U, King V, Miettunen J, Ek E, Laitinen J (2007). Cloninger's Temperament Dimensions, Socio-economic and Lifestyle Factors and Metabolic Syndrome Markers at Age 31 Years in the Northern Finland Birth Cohort 1966.. J Health Psychol.

[36] Lindstrom ML, Bates DM (1990). Nonlinear mixed effects models for repeated measures data.. Biometrics.

[37] Jolicoeur P, Pontier J, Pernin MO, Sempe M (1988). A lifetime asymptotic growth curve for human height.. Biometrics.

