# Supplementary material for: Genetic Determinants of Height Growth Assessed Longitudinally from Infancy to Adulthood in the Northern Finland Birth Cohort 1966
Source: PLoS Genet. 2009 Mar 6;5(3):e1000409. doi: 10.1371/journal.pgen.1000409 (PMC2646138; doi:10.1371/journal.pgen.1000409)
Supplement: Table S1 — Spearman correlation coefficient between growth parameters and growth measures at birth and in adulthood. (0.06 MB DOC) [file pgen.1000409.s001.doc]

Table S1. Spearman correlation coefficient between growth parameters and growth measures at birth and in adulthood.

| **FEMALE** | PHV1 | PHV2 | ATO | Age at PHV2 | BW | BL | GA | Height | BMI |
| --- | --- | --- | --- | --- | --- | --- | --- | --- | --- |
| PHV1 | 1 |  |  |  |  |  |  |  |  |
| PHV2 | 0.078 | 1 |  |  |  |  |  |  |  |
| ATO | -0.061 | -0.812 | 1 |  |  |  |  |  |  |
| Age at PHV2 | -0.070 | -0.590 | 0.900 | 1 |  |  |  |  |  |
| BW | -0.059 | -0.032 | 0.108 | 0.078 | 1 |  |  |  |  |
| BL | -0.058 | -0.008 | 0.096 | 0.065 | 0.774 | 1 |  |  |  |
| GA | -0.051 | 0.002 | 0.009 | 0.013 | 0.341 | 0.343 | 1 |  |  |
| Height | 0.449 | 0.140 | 0.097 | 0.041 | 0.259 | 0.331 | -0.001 | 1 |  |
| BMI | -0.017 | -0.027 | -0.101 | -0.169 | 0.056 | 0.000 | 0.003 | -0.112 | 1 |
| Agemen | -0.056 | -0.349 | 0.523 | 0.575 | 0.025 | 0.034 | -0.012 | 0.106 | -0.200 |
| **MALE** |  |  |  |  |  |  |  |  |  |
| PHV1 | 1 |  |  |  |  |  |  |  |  |
| PHV2 | 0.119 | 1 |  |  |  |  |  |  |  |
| ATO | -0.094 | -0.861 | 1 |  |  |  |  |  |  |
| Age at PHV2 | -0.098 | -0.748 | 0.969 | 1 |  |  |  |  |  |
| BW | -0.085 | -0.017 | 0.032 | 0.017 | 1 |  |  |  |  |
| BL | -0.050 | 0.007 | 0.010 | -0.011 | 0.786 | 1 |  |  |  |
| GA | -0.063 | -0.032 | 0.007 | -0.002 | 0.402 | 0.382 | 1 |  |  |
| Height | 0.460 | 0.088 | 0.007 | -0.047 | 0.261 | 0.347 | 0.037 | 1 |  |
| BMI | 0.002 | 0.031 | -0.150 | -0.190 | 0.052 | 0.038 | 0.041 | -0.030 | 1 |

Reed1 model: PHV1 = peak height velocity in infancy; JPA-2 model: PHV2 = peak height velocity in puberty, ATO = age at height growth spurt take-off, Age at PHV2 = age at peak height velocity in puberty; BW = birth weight, BL = birth length, GA = gestational age, Height is measured at age 31 years, BMI = body mass index at age 31 years, Agemen = age at menarche in females.
