# Supplementary material for: Genetic Determinants of Height Growth Assessed Longitudinally from Infancy to Adulthood in the Northern Finland Birth Cohort 1966
Source: PLoS Genet. 2009 Mar 6;5(3):e1000409. doi: 10.1371/journal.pgen.1000409 (PMC2646138; doi:10.1371/journal.pgen.1000409)
Supplement: Table S2 — Summary of the SNPs genotyped in the NFBC1966. (0.26 MB DOC) [file pgen.1000409.s002.doc]

Table S2. Summary of the SNPs genotyped in the NFBC1966.

| **SNP** | **Original reference for SNP** | **Gene abbreviation, name** | **SNP location** | **Chromosomal Position^1^** | **HapMap Alleles + strand** | **HapMap MAF^2^** | **NFBC1966 MAF^2^** | **HWE P** | **GSR**^5^ |
| --- | --- | --- | --- | --- | --- | --- | --- | --- | --- |
| rs11205277 | [5] | *SF3B4/SV2A* , between splicing factor 3b, subunit 4, 49kDa and synaptic vesicle glycoprotein 2A | Between genes | Chr1:146705945 | A/G | G_0.39 | G_0.31 | 0.427 | 99.9 |
| rs6830062 | [5] | *LCORL* , ligand dependent nuclear receptor corepressor-like | Between genes | Chr4:17693999 | T/C | C_0.16 | C_0.09^3^ | 0.560 | 99.9 |
| rs3116602 | [4] | *DLEU7,* deleted in lymphocytic leukemia, 7 | Within gene | Chr13:50009356 | T/G | G_0.18 | G_0.30 | 0.033 | 93.8 |
| rs4713858 | [5] | *PPARD/ FANCE* , between peroxisome proliferator-activated receptor delta and Fanconi anemia, complementation group E | Between genes | Chr6:35510763 | G/A | A_0.16 | A_0.11 | 0.832 | 100 |
| rs10946808 | [6] | *HIST1H1D*, downstream of histone cluster 1, H1d | Between genes | Chr6:26341366 | A/G | G_0.28 | G_0.42 | 0.034 | 99.9 |
| rs6854783 | [4], also proxy for rs1812175 [5] and for rs1492820 [6] | *HHIP* , hedgehog interacting protein | Within gene | Chr4:146000684 | A/G | G_0.45 | G_0.35 | 0.936 | 95.1 |
| rs6060373 | [4], also proxy for rs6060369 [6] | *UQCC,* ubiquinol-cytochrome c reductase complex chaperone | Within gene | Chr20:33377622 | A/G | G_0.36 | G_0.43 | 0.936 | 97.7 |
| rs6724465 | [4] | *NHEJ1,* nonhomologous end-joining factor 1 | Within gene | Chr2:219769351 | G/A | A_0.16 | A_0.08 | 0.682 | 89.0 |
| rs2814993 | [4] | *C6orf106,* chromosome 6 open reading frame 106 | Within gene | Chr6:34726871 | G/A | A_0.10 | A_0.20 | 0.284 | 94.3 |
| rs6842303 | [5] | *LCORL* , ligand dependent nuclear receptor corepressor-like | Within gene | Chr4:17530324 | G/T | T_0.33 | T_0.28^3^ | 0.832 | 99.9 |
| rs11107116 | [4] | *SOCS2,* suppressor of cytokine signaling 2 | Within gene | Chr12:92480972 | G/T | T_0.20 | T_0.25 | 0.445 | 94.6 |
| rs12459350 | Proxy for rs12986413 [6] | *DOT1L* , DOT1-like, histone H3 methyltransferase (S. cerevisiae) | Within gene | Chr19:2127586 | A/G | G_0.45 | G_0.45 | 0.647 | 99.9 |
| rs4800148 | [5] | *CABLES1,* Cdk5 and Abl enzyme substrate 1 | Within gene | Chr18:18978326 | A/G | G_0.26 | G_0.23 | 0.576 | 99.9 |
| rs2562785 | Proxy for rs2562784 [6] | *SH3GL3,* SH3-domain GRB2-like 3 | Within gene | Chr15:82077025 | G/T | T_0.15 | T_0.14 | 0.454 | 99.6 |
| rs4794665 | [5] | *C17orf67,* upstream of chromosome 17 open reading frame 67 | Between genes | Chr17:52205328 | A/G | G_0.48 | A_0.48 | 0.977 | 99.9 |
| rs4549631 | [4] | *C6orf173,* downstream of chromosome 6 open reading frame 173 | Between genes | Chr6:127008001 | T/C | C_0.43 | C_0.46 | 0.593 | 94.7 |
| rs7846385 | [5] | *PXMP3/ PKIA*, between peroxisomal membrane protein 3, 35kDa and protein kinase (cAMP-dependent, catalytic) inhibitor alpha | Between genes | Chr8:78322734 | T/C | C_0.34 | C_0.22^3^ | 0.262 | 99.7 |
| rs1042725 | [4], also proxy for rs8756 [5], also [6] | *HMGA2,* high mobility group AT-hook 2 | Within gene | Chr12:64644614 | C/T | T_0.42 | T_0.50 | 0.526 | 97.9 |
| rs4533267 | [5] | *ADAMTS17,* ADAM metallopeptidase with thrombospondin type 1 motif, 17 | Within gene | Chr15:98603794 | G/A | A_0.28 | A_0.24 | 0.001 | 99.9 |
| rs3731343 | Proxy for rs2040494 [6] | *CDK6,* cyclin-dependent kinase 6 | Within gene | Chr7:91918187 | A/C | C_0.49 | A_0.49 | 0.910 | 99.9 |
| rs314277 | [6] | *LIN28B*, lin-28 homolog B (C. elegans) | Within gene | Chr6:105514355 | C/A | A_0.13 | A_0.17 | 0.267 | 98.6 |
| rs8041863 | [4] | *ACAN,* aggrecan | Within gene | Chr15:87160693 | T/A | A_0.49 | A_0.37^3^ | 0.650 | 98.1 |
| rs12735613 | [4] | *SPAG17*, sperm associated antigen 17 | Within gene | Chr1:118596015 | G/A | A_0.31 | A_0.24 | 0.183 | 97.6 |
| rs10935120 | [4] | *CEP63,* centrosomal protein 63kDa | Within gene | Chr3:135715790 | G/A | A_0.36 | A_0.25 | 0.333 | 96.9 |
| rs10906982 | [4] | *ADAMTSL3,* ADAMTS-like 3 | Within gene | Chr15:82359162 | T/A | A_0.48 | A_0.48^3^ | 0.617 | 96.0 |
| rs10512248 | [4] | *PTCH1,* patched homolog 1 (Drosophila) | Within gene | Chr9:95339258 | T/G | G_0.32 | G_0.38 | 0.417 | 98.3 |
| rs6440003 | [4], also proxy for rs6763931 [5] and for rs724016 [6] | *ZBTB38,* zinc finger and BTB domain containing 38 | Within gene | Chr1:41199964 | G/A | A_0.48 | A_0.46 | 0.062 | 94.0 |
| rs6686842 | [4] | *SCMH1,* sex comb on midleg homolog 1 (Drosophila) | Within gene | Chr1:41199964 | C/T | T_0.41 | T_0.41 | 0.838 | 94.4 |
| rs3791675 | [4], also proxy for rs3791679 [5] | *EFEMP1,* EGF-containing fibulin-like extracellular matrix protein 1 | Within gene | Chr2:56022960 | C/T | T_0.28 | T_0.24 | 0.221 | 97.9 |
| rs2282978 | [4], also [5] | *CDK6,* cyclin-dependent kinase 6 | Within gene | Chr7:91909061 | T/C | C_0.37 | C_0.31 | 0.413 | 95.2 |
| rs9650315 | [4], also [6] | *CHCHD7,* coiled-coil-helix-coiled-coil-helix domain containing 7 | Within gene | Chr8:57318152 | G/T | T_0.13 | T_0.13 | 0.198 | 95.1 |
| rs8007661 | Proxy for rs1390401 [4], also [6] | *TRIP11,* thyroid hormone receptor interactor 11 | Within gene | Chr14:91529711 | C/T | T_0.30 | T_0.48 | 0.249 | 95.9 |
| rs678962 | [5] | *DNM3,* dynamin 3 | Within gene | Chr1:168921546 | T/G | G_0.16 | G_0.23^3^ | 0.937 | 99.9 |
| rs7153027 | [5] | *TRIP11/FBLN5,* between thyroid hormone receptor interactor 11 and fibulin 5 | Between genes | Chr14:91496975 | A/C | C_0.39 | C_0.41 | 0.907 | 99.6 |
| rs3760318 | [5] | *ADAP2,* upstream of ArfGAP with dual PH domains 2 | Between genes | Chr17:26271841 | G/A | A_0.36 | A_0.40 | 0.393 | 99.9 |
| rs757608 | [5] | *TBX2,* T-box 2 | Within gene | Chr17:56852059 | G/A | A_0.30 | A_0.26 | 0.001 | 99.6 |
| rs967417 | [5] | *BMP2,* bone morphogenetic protein 2 | Within gene | Chr20:6568893 | G/A | A_0.43 | G_0.44 | 0.388 | 99.9 |
| rs12198986 | [5] | *BMP6,* Upstream of bone morphogenetic protein 6 | Within gene | Chr6:7665058 | A/G | A_0.50 | A_0.47 | 0.460 | 99.8 |
| rs2844479 | [5] | *RDBP, (LST1) NCR3/AIF1,* within RD RNA binding protein, between (leukocyte specific transcript 1), natural cytotoxicity triggering receptor 3 and allograft inflammatory factor 1^4^ | Between genes | Chr6:31680935 | A/C | C_0.32 | C_0.28 | 0.295 | 100 |
| rs3130050 | [5] | *RDBP/BAT3,* between RD RNA binding protein and HLA-B associated transcript 3 | Between genes | Chr6:31726740 | A/G | G_0.23 | G_0.14 | 0.953 | 99.9 |
| rs185819 | [5] | *TNXB*, tenascin XB | Within gene | Chr6:32158045 | T/C | C_0.43 | T_0.48^3^ | 0.364 | 99.9 |
| rs1776897 | [5] | *HMGA1,* upstream of high mobility group AT-hook 1 | Within gene | Chr6:34302989 | T/G | G_0.08 | G_0.06 | 0.115 | 99.9 |
| rs6570507 | Proxy for rs4896582 [6] | *GPR126,* G protein-coupled receptor 126 | Within gene | Chr6:142721265 | G/A | A_0.25 | A_0.26 | 0.325 | 99.9 |
| rs3748069 | [5], also proxy for rs4896582 [6] | *GPR126,* downstream of G protein-coupled receptor 126 | Between genes | Chr6:142809326 | A/G | G_0.27 | G_0.26 | 0.259 | 99.9 |
| rs798544 | [5] | *AMZ1/GNA12,* between archaelysin family metallopeptidase 1 and guanine nucleotide binding protein (G protein) alpha 12 | Between genes | Chr7:2536343 | C/T | T_0.28 | T_0.37 | 1.000 | 99.9 |
| rs11765954 | [5] | *CDK6,* cyclin-dependent kinase 6 | Within gene | Chr7:91925346 | T/C | C_0.29 | C_0.25^3^ | 0.165 | 99.9 |
| rs10958476 | [5] | *PLAG1,* pleiomorphic adenoma gene 1 | Within gene | Chr8:57258362 | T/C | C_0.13 | C_0.22^3^ | 0.647 | 99.8 |
| rs4743034 | [5] | *ZNF462,* zinc finger protein 462 | Within gene | Chr9:106711908 | G/A | A_0.24 | A_0.20 | 0.593 | 99.9 |

^1^Chromsomal positions are from the NCBI Build 35, produced by the International Human Genome Sequencing Consortium. ^2^Allele frequency is for the minor allele observed in the HapMap CEU population, or the NFBC1966 population, which differ in some instances. ^3^Genotyped on the negative strand. ^4^*RDBP* spans the region Chr6:31529510-32034664, within that region *NCR3* and *AIF1* are also encoded, *LST1* is also overlapping with *RDBP* and *NCR3*. ^5^GSR = genotyping success rate. Duplication was carried out for all genotyping carried out in-house, most duplication error rates were <0.1% (rs807661 was 1.1%, however the duplication plate had a low genotyping success rate overall).
