# Supplementary material for: Genetic Determinants of Height Growth Assessed Longitudinally from Infancy to Adulthood in the Northern Finland Birth Cohort 1966
Source: PLoS Genet. 2009 Mar 6;5(3):e1000409. doi: 10.1371/journal.pgen.1000409 (PMC2646138; doi:10.1371/journal.pgen.1000409)
Supplement: Text S1 — Description of growth modelling methods, derived growth parameters and their correlations with other growth measures. (0.05 MB DOC) [file pgen.1000409.s003.doc]

*Supporting Text*

## Height growth curve modelling

Parametric growth curves were fitted separately for boys and girls using nonlinear random effects models. The best-fitting curves for each individual were estimated by maximum likelihood method. Height growth velocity curves were obtained as first derivatives and acceleration curves as second derivatives of the height growth curves. Velocity and acceleration curves were then used to draw biologically meaningful parameters - peak height velocities and puberty timing indicators - as described below. Mean-constant curves [8] that describe the average pattern of height growth velocity in the population were drawn for illustrative purpose for certain SNPs by genotype and sex using the estimated model parameters (see Figure 1). The nlme function in R program (version 2.4.1) was used to fit the models. It applies the model formulation and computational methods described in Lindstrom and Bates [36].

**Early growth**

The fit of five different growth models [17] for early growth (age 0-24 months) were compared in terms of residual standard deviation (RSD) and Akaike Information Criterion (AIC). The Reed1 model [32] was chosen since it showed a better fit to the early height growth data than the Kouchi, Carlberg and Count models, and an equally good fit to the Reed2 model which has one more parameter than the Reed1 model.

**Reed1 model**

The Reed1 model is a 4-parameter extension of the 3-parameter Count model [9] and its functional form is

*Y* = *A* + *Bt* + *C*ln(*t*) + *D*/*t*

[32]. Since this model is not defined at birth (t=0), it was modified for this study in the same way as in Simondon et al [17]:

*Y* = *A* + *Bt* + *C*ln(*t*+1) + *D*/(*t*+1), where

*t* = postnatal age

*Y* = height reached at age *t*

and *A*, *B*, *C* and *D* the function parameters.

Of the function parameters, *A* is related to the baseline height at birth, *B* to the linear component of the growth velocity, *C* to the decrease in the growth velocity over time, and *D* to the inflection point that allows growth velocity to peak after birth rather than exactly at birth. The Reed1 model is linear in its constants [9].

All subjects with at least one height measurement from birth to 24 months at least 0.1 month (~3 days) apart were included in the Reed1 model fitting. Although the model converged for the whole group, random effects for parameters b and c were estimated to be zero for those with only one measurement (typically birth length). Even having two measurements was inadequate to capture the shape of the growth curve and therefore we restricted the SNP association analysis to those with a minimum of three measurements per person.

The parameters derived from the fitted Reed1 models were PHV in infancy (PHV1), lowest height velocity in infancy (LHV1), and mean height velocity in infancy (MEANHV1). Due to high correlations between these three variables (0.81-0.99 in both sexes), results are reported only on PHV1.

**Pubertal growth**

To obtain parameters for puberty, JPPS and JPA-2 models [33] were fitted for the whole growth period from birth to adulthood (0-20 years) and due to a better fit the parameters from the JPA-2 model were selected for further analyses.

Due to the high number of parameters in the JPA-2 model and sparseness of measurements after infancy in the data, the sample had to be restricted to those with at least 16 height measurements between 0 and 20 years at least 0.1 month (~3 days) apart to reach model convergence.

**JPA-2 model**

The parametric JPA-2 model can be written as

*Y* = *A*[1 – [1+ ((*t*+*E*)/*D1*)*^C1^* + ((*t*+*E*)/*D2*)*^C2^* + ((*t*+*E*)/*D3*)*^C3^*]^-1^], where

*t* = postnatal age

*Y* = height reached at age *t*

*A* = adult height

*E* = estimated prenatal duration of growth

*D1*, *D2*, *D3* = time-scale factors

*C1*, *C2*, *C3* = dimensionless exponents

All parameters are assumed to be nonnegative.

The JPA-2 model includes eight parameters. It is an extension of the seven-parameter JPPS model which includes three components that match with natural periods of human growth: infancy, early childhood and puberty [37]. JPA-2 estimates the prenatal duration of growth (*E*), whereas JPPS fixes it at the average gestational age (*E*=0.75 years). This does not take into account individual variation in the gestational age or the presumed delay after conception before the start of the growth in stature [33]. Therefore the extension from JPPS to JPA-2 improves the model fit in infancy. JPA-2 has been found to fit human growth data better than several other suggested models [18,33]. PHV in puberty (PHV2) was derived from the individual velocity curve and age at height growth spurt “take-off” (ATO) and age at PHV2 from the acceleration curve estimated from the JPA-2 model. The maximum height growth velocity reached at age 8-16y in girls and 9-17 in boys was defined as PHV2. ATO was defined to be the age ≥8 years in girls and ≥9 years in boys when acceleration first changed its sign from negative to positive. Age at PHV2 was defined to be the age when it subsequently changed its sign from positive to negative.

In summary, the growth parameters derived from the JPA-2 model were PHV1, PHV2, ATO and age at PHV2. Since PHV1 can be derived for a higher number of people from the Reed1 model with the added flexibility on the timing of early velocity peak, only SNP associations on puberty parameters are reported from the JPA-2 model.

Quality checks for derived variables

The mutual correlations between derived growth parameters and their correlations with birth measures (birth weight, birth length and gestational age), adult height and BMI were calculated by sex; Spearman correlation coefficients are reported (Table S1). Additionally in girls, the correlations between growth parameters and self-reported age at menarche were assessed. SAS (version 9.1.3.) was used for these analyses.

Reed1 model results

PHV1 was derived for N=2115 boys and N=2110 girls, before exclusion of those without genetic data. The means and standard deviations are reported in Table 1. The distributions were slightly skewed to the right and therefore the variable was log-transformed for the genotype association analysis.

As expected, PHV1 had a weak negative correlation (from -0.05 to -0.08) with all birth measures (0.0001<p<0.02 in both sexes) and a strong positive correlation with adult height (Table S1). There was no correlation between PHV1 and adult BMI (p>0.4 in both sexes). Girls who grew faster in infancy had a slightly lower age at menarche (p=0.011).

JPA-2 model results

Pubertal growth parameters were successfully derived for N=1533 females and 1573 males from the JPA-2 model, before exclusion of those without genetic data. ATO and age at PHV2 were normally distributed but PHV2 was slightly skewed to the right and was log-transformed for further analysis. The means and standard deviations of these parameters before transformations are reported in Table 1.

Those with late pubertal height growth spurt had the lowest growth velocity in puberty: the correlation between ATO / age at PHV2 and PHV2 varied between -0.59 and -0.86 (Table S1). ATO and age at PHV2 were highly correlated with each other..

As PHV1 from the JPA-2 model was highly correlated with PHV1 from the Reed1 model (coefficients 0.80 and 0.79 in boys and girls, respectively), its correlations with birth measures and adult body size are not reported here. In boys, there were no correlations between the birth measures and any of the three growth parameters at puberty from the JPA-2 model (Table S1). In girls, higher birth weight and birth length were associated with slightly higher ATO and age at PHV2 (coefficients 0.07-0.11, p between <0.0001 and 0.011). As expected, PHV2 was positively correlated with adult height (p<0.0001 in girls and p=0.0005 in boys). ATO was positively correlated with adult height in girls (coefficient 0.10, p=0.0001) but not in boys (coefficient 0.007, p=0.78) but age at PHV2 was not associated with adult height in either sex. Both ATO and age at PHV2 were inversely correlated with adult BMI in both sexes (coefficients between -0.10 and -0.19, p<0.0001 in all). In girls, a higher age of menarche was strongly correlated with higher ATO and age at PHV2 and lower PHV2.
